# FASTER and SCOTT&EVA trainings for adults with high-functioning autism spectrum disorder (ASD): study protocol for a randomized controlled trial

**DOI:** 10.1186/s13063-021-05205-9

**Published:** 2021-04-08

**Authors:** Ludger Tebartz van Elst, Thomas Fangmeier, Ulrich Max Schaller, Oliver Hennig, Meinhard Kieser, Katja Koelkebeck, Charlotte Kuepper, Veit Roessner, Dirk Wildgruber, Isabel Dziobek

**Affiliations:** 1 Department of Psychiatry and Psychotherapy Medical Center, University of Freiburg Faculty of Medicine, Freiburg im Breisgau, Germany; 2 Department of Child and Adolescent Psychiatry, Psychotherapy, and Psychosomatics, Medical Center, University of Freiburg Faculty of Medicine, Freiburg im Breisgau, Germany; 3 Central Institute for Mental Health, Mannheim, Germany; 4 Institute of Medical Biometry and Informatics, University Hospital Heidelberg, Heidelberg, Germany; 5 Department of Psychiatry and Psychotherapy, LVR-Hospital Essen, Medical Faculty, University of Duisburg-Essen, Duisburg, Germany; 6 Department of Psychology, Berlin School of Mind and Brain, Humboldt-Universitaet zu Berlin, Berlin, Germany; 7 Department of Child and Adolescent Psychiatry and Psychotherapy, Faculty of Medicine, TU Dresden, Dresden, Germany; 8 Department of Psychiatry and Psychotherapy, University of Tuebingen, Tübingen, Germany

**Keywords:** Adults, Autism spectrum disorder, Psychotherapy, High-functioning autism, Internet-based training, Social cognition training, Social skills intervention, Randomized controlled trial

## Abstract

**Background:**

Autism spectrum disorder (ASD) is a chronic neurodevelopmental condition with a prevalence rate above 1%, characterized by deficits in social communication and interaction; restrictive, repetitive patterns of behavior, interests, or activities; and a preference for sameness and routines. The majority of adult ASD patients suffer from comorbid conditions such as depression and anxiety. Therapy options for adult ASD patients are lacking, with presently no available evidence-based interventions in Germany. Recently, two interventions to improve social responsiveness have been published. FASTER (“Freiburger Asperger-Spezifische Therapie für ERwachsene” = Freiburg Asperger-specific therapy for adults) is a manualized group psychotherapy program including three modules on psychoeducation, stress regulation management, and non-verbal and verbal social communication training with videotaped tasks. SCOTT&EVA (“Social Cognition Training Tool”, and its enhancement “Emotionen Verstehen und Ausdruecken” = understanding and expressing emotions) is a computer-based training program to enhance social cognition including video and audio material of emotional expressions and complex real-life social situations. Initial studies for both programs have shown good feasibility and efficacy.

**Methods:**

Three hundred sixty adult participants with an autism spectrum disorder (ASD) will take part in a randomized controlled three-armed multi-center trial to prove the efficacy of manualized group psychotherapy and a manualized computer-based training program. Both interventions will be compared with a treatment as usual (TAU) group, aiming to establish evidence-based psychotherapy approaches for adult individuals with ASD. The primary outcome is evaluated by parents, spouses, or others who have sufficient insight into the respective participant’s social communication and interaction, and will be measured with the Social Responsiveness Scale. First, each of both interventions will be compared to TAU. If at least one of the differences is significant, both interventions will be compared against each other. The primary outcome will be measured at baseline (T0) and 4 months after baseline (T1).

**Discussion:**

The trial is the first to validate psychiatric therapeutic and training interventions for adult ASD patients in Germany. A trial is needed because the prevalence of ASD in adulthood without intellectual disability is high, and no evidence-based intervention can be offered in Germany.

**Trial registration:**

German Clinical Trial Register DRKS00017817. Registered on 20 April 2020.

## Administrative information

**Table:** 

Title {1}	Evaluation of a three-arm, cluster-randomized controlled study to assess the effects of FASTER and SCOTT&EVA trainings for adults with high-functioning autism spectrum disorder (ASD) Trial acronym: FASTER/SCOTT Study
Trial registration {2a and 2b}.	German Clinical Trial Register, DRKS00017817, Registered 20 April 2020.
Protocol version {3}	Issue Date: 3 July 2020 Trial protocol version: 1.94 Authors: LTvE, TF, UMS, OH, MK, KK, CK, VR, DW, ID
Funding {4}	German Research Foundation (DFG TE 280/18-1; https://gepris.dfg.de/gepris/person/1462933; Deutsche Forschungsgemeinschaft)
Author details {5a}	Ludger Tebarzt van Elst (Corresponding Author, shared first authorship) Department of Psychiatry and Psychotherapy Medical Center – University of Freiburg, Faculty of Medicine University of Freiburg, Germany Thomas Fangmeier (shared first authorship) Department of Psychiatry and Psychotherapy Medical Center – University of Freiburg, Faculty of Medicine University of Freiburg, Germany Ulrich Max Schaller Department of Psychiatry and Psychotherapy Medical Center – University of Freiburg, Faculty of Medicine University of Freiburg, Germany Department of Child and Adolescent Psychiatry, Psychotherapy, and Psychosomatics; Medical Center, University of Freiburg, Faculty of Medicine University of Freiburg Oliver Henning Central Institute for Mental Health, Mannheim Meinhard Kieser Institute of Medical Biometry and Informatics, University Hospital Heidelberg Katja Koelkebeck LVR-Hospital Essen, department of Psychiatry and Psychotherapy, Medical Faculty, University of Duisburg-Essen Charlotte Kuepper Berlin School of Mind and Brain, Department of Psychology, Humboldt-Universitaet zu Berlin Veit Roessner Department of Child and Adolescent Psychiatry and Psychotherapy, Faculty of Medicine, TU Dresden Dirk Wildgruber Department of Psychiatry and Psychotherapy, University of Tuebingen Isabel Dziobek Berlin School of Mind and Brain, Department of Psychology, Humboldt-Universitaet zu Berlin
Name and contact information for the trial sponsor {5b}	**Trial Sponsor:** Department of Psychiatry and Psychotherapy, Medical Center – University of Freiburg, Faculty of Medicine, University of Freiburg, Germany **Contact name:** Prof. Dr. med. Ludger Tebartz van Elst **Address:** Medical Center – University of Freiburg, Department of Psychiatry and Psychotherapy, Hauptstrasse 5, 79104 Freiburg, Germany **E-Mail:** tebartzvanelst@uniklinik-freiburg.de
Role of sponsor {5c}	The reviewers of the German Research Foundation had impact on the original study design. Per request of the reviewers, randomization has been changed from 4:3:3 (FASTER : SCOTT&EVA : TAU) to 1:1:1, resulting in a higher sample size than initially intended. Further, reviewers requested the Social Responsiveness Scale (external assessment) as primary outcome measure.

## Introduction

### Background and rationale {6a}

Autism spectrum disorders (ASD) are neurodevelopmental conditions behaviorally characterized by impairments in social communication and interaction as well as the presence of repetitive and restricted patterns of behaviors, interests, and activities [1, 2]. While the topic is well established in Child and Adolescent Psychiatry and Psychotherapy, this is not yet the case in Adult Psychiatry and Psychotherapy (APP), although the current prevalence rate with 1–2% of ASD in the general population [2, 3] is high, as ASD is a lifelong condition, in which symptoms do not simply disappear.

In past clinical practice, ASD in adulthood has was regarded as a neurodevelopmental disorder, which generally goes along with overt and severe language deficits, learning problems and low IQ. Only few adult high-functioning patients received the diagnosis ASD, but instead were often been diagnosed only by their secondary psychiatric conditions [4]. In these constellations, however, ASD without intellectual disability can be understood as a kind of basic disorder from which typical psychosocial consequences, communicatory problems, and interpersonal conflicts arise. These in turn often evoke secondary affective and other psychiatric disorders [5].

Adults with ASD show high rates of psychiatric comorbidity such as depression (53%), anxiety (50%), attention deficit hyperactivity disorder (43%), obsessive-compulsive disorder (24%), tic disorders (20%), and psychotic disorders (12%) [4, 6–8]. This results in a lifetime consultation rate of psychiatric services of 78% [9]. Furthermore, rates of suicidality are higher than in the general population [10, 11]. The majority of adults with ASD show a long-lasting psychiatric history.

Given the deficits in social cognition and social interaction, individuals with ASD often have significant interpersonal problems. Difficulties in understanding pragmatic aspects of language frequently lead to social withdrawal, depression, and anxiety. This may result in intensified social withdrawal, isolation, and unemployment [3]. Unemployment in this context is not associated with a lack of professional competence, but with communication difficulties with superiors and peers as well as difficulties to find the correct tune between accuracy and working speed. Adults diagnosed with ASD later in their life often show high formal qualifications in contrast to high rates of unemployment, early retirement, and overqualification for actual jobs [12]. The need to permanently apply cognitive coping strategies during social interactions can lead to perceptive, attentional, and emotional overload, implicating difficulties to cope with additional co-occurring stressful situations [9].

In summary, these results show that there is a need (i) to implement valid diagnostic investigation of adults with symptoms of ASD in the diagnostic routines of APP and (ii) to develop specific psychotherapy programs addressing the core features of ASD. Presently, such therapy options for adult patients are rare [2, 5, 9]. To counteract this problem, two therapeutic approaches were developed by research groups of the Department of Psychiatry and Psychotherapy, Faculty of Medicine, University of Freiburg and the Berlin School of Mind and Brain, Humboldt-Universitaet zu Berlin, aiming to improve social responsiveness in adults with ASD. The Freiburg Asperger-specific therapy for adults (German: Freiburger Autismus-Spezifische Therapie für ERwachsene, FASTER) was developed at the Department of Psychiatry and Psychotherapy in Freiburg. The Social Cognition Training Tool (SCOTT&EVA; EVA English: Understanding and Expressing Emotion, German: Emotionen Verstehen und Ausdruecken) was developed at the Berlin School of Mind and Brain, Humboldt-Universitaet zu Berlin. Both intervention programs address the core symptoms of high-functioning ASD, such as deficits in social perception, categorization, and communication, as well as deficits in emotion regulation in everyday situations. The main objectives of both are to improve social responsiveness and social competence. The FASTER intervention is a specific manualized group psychotherapy for the treatment of adult patients with high-functioning ASD [13] to improve social responsiveness and social cognition by including psychoeducational aspects and stress management in addition to teaching social conversational skills. The SCOTT intervention is a computer-based, manualized training program that can be used via an Internet-portal platform to target social cognitive impairments of high-functioning adults with ASD by fostering the recognition of different emotions from facial expressions, prosody, and complex social situations [14]. The original SCOTT intervention has been extended to SCOTT&EVA to include more varied training modules and an adaptive training mechanism that adjusts training difficulty to a user’s performance level [15].

To advance the process of empirical validation of these intervention methods, we designed a combined controlled, randomized trial for both treatment approaches, which has received funding from the German Research Foundation.

### Objectives {7}

The main objective of this trial is to prove the effectiveness and efficacy of the therapy programs FASTER and SCOTT&EVA in a combined phase-III trial.

The primary hypothesis is if there is superiority of at least one of the treatments FASTER and SCOTT&EVA over treatment as usual (TAU) regarding social responsiveness.

If this is confirmed, the difference between FASTER and SCOTT&EVA regarding the primary endpoint will be tested confirmatorily. Otherwise, the comparison between FASTER and SCOTT&EVA will be done descriptively.

### Trial design {8}

**Fig. 1:**
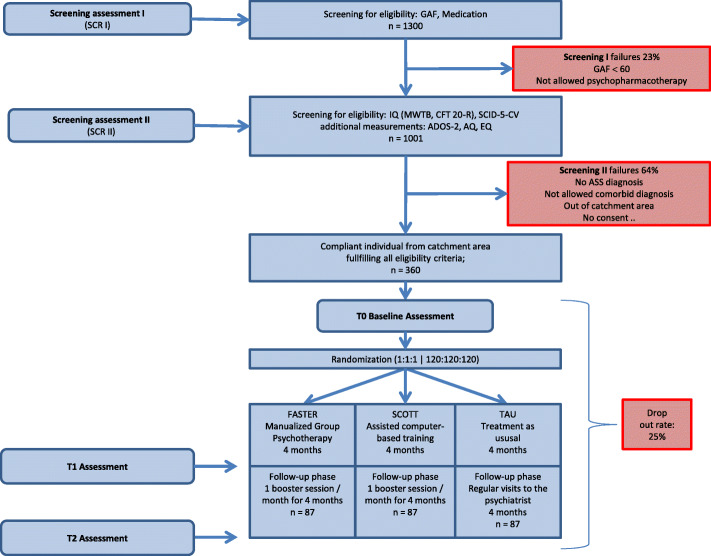
Illustration of the trial flow

This trial is a controlled, three-armed, cluster-randomized, observer-blinded, multi-center study to compare FASTER and SCOTT&EVA with TAU as parallel groups (see Fig. 1). The allocation ratio will be 1:1:1.

## Methods: participants, interventions, and outcomes

### Study setting {9}

The comparison between the FASTER and SCOTT&EVA interventions and TAU will be implemented as a national interventional multi-center trial at six centers in Germany. Six centers in Germany take part in the recruitment and implementation of the study. The list of recruiting study sites can be obtained from the German Clinical Trial Register with the register number DRKS00017817 (internet site: https://www.drks.de/drks_web/navigate.do?navigationId=trial. HTML&TRIAL_ID=DRKS00017817).

### Eligibility criteria {10}

The trial sample will comprise 360 participants. Both women and men are included into the study. Participants will be randomly assigned to one of the three groups in a cluster-randomized manner.

The number of participants is needed to detect intervention-related differences with the desired power.

#### Inclusion criteria

Participation is possible in case of one of the following diagnoses:

Asperger syndrome (ICD-10: F84.5; DSM-IV-TR: 299.80), high-functioning autism (ICD-10: F84.0; DSM-IV-TR: 299.00), or atypical autism, if the DSM-IV criterion A and deficits in social communication and social interaction are fulfilled (ICD-10: F84.1; DSM-IV-TR: 299.00). All three diagnoses are current standard diagnoses in Germany, encoded with the F-keys from the International Statistical Classification of Diseases and Related Health Problems, Book V (ICD-10, [16]). Equivalent codes for the Diagnostic and Statistical Manual of Mental Disorders (DSM-IV-TR, [17]) are also reported. The DSM-IV criteria are better suited to describe the equivalent ICD-10 criteria currently used in Germany than the more recent version of the Diagnostic and Statistical Manual (DSM-5, [1]), which has significant changes.

Further inclusion criteria for our study are an age between 18 and 65 years, an IQ higher or equal 80, relevant psychosocial impairment lower or equal to a score of 60 as measured with the Global Assessment of Functioning [1], the ability to understand the character and individual consequences of the trial, written informed consent, consent for audio recordings of therapy sessions, ability to sufficiently communicate in groups, no severe reading or writing disability, none or stable psychopharmacotherapy, fluent German language of study participant, and one other person who is able to fill out the questionnaire for social responsiveness [18, 19].

#### Exclusion criteria

Psychiatric comorbidities for exclusion are as follows: florid schizophrenia (ICD-10: F20.X; DSM-IV-TR: 295.xx, 298.9), florid psychosis (ICD-10: F23.X, F24; DSM-IV-TR: 297.1, 297.3, 298.8), acute manic episode within a bipolar disorder (ICD-10: F30.X, F31.X, F34.0; DSM-IV-TR: 296.4x, 296.5x, 296.6x, 296.80, 296.89, 296.90, 301.13, 300.4), acute severe depression (ICD-10: F32.2, F32.3, DSM-IV-TR: 296.23, 296.24), or acute suicidality. Further, substance abuse or substance dependencies within the last 12 months (ICD-10; F1X; DSM-IV-TR: 291.0, 291.8, 292.0, 292.81, 292.89, 303.00, 303.90, 304.x, 305.90, 305.x), present or past gambling disorder, not corrected severe vision or hearing impairment, a history of severe group disturbing behavior (documented therapy exclusions from previous group therapies), any neurological disorder or medical condition interfering with group therapy, group-based social skills training or other structured psychotherapy for the core symptoms of ASD (current or in the last 6 months prior to the study, structured psychotherapy for comorbid disorders like depression is allowed), inpatient treatment at baseline (T0) is also an exclusion criterion; however, inclusion is possible after the completion of inpatient treatment.

### Who will take informed consent? {26a}

Patients will receive information on the content and process of the study by mail and will be asked to give preliminary consent for data storage if they are interested in the study. In the next step, potential participants will be contacted by phone or invited into the study center for a first screening interview.

This first interview only comprises some short questions about eligibility. It will be performed by trained study personnel. If no exclusion criterion is applicable, the participants will be invited to come to a face-to-face interview to the study center. During the second interview, trained study staff (physicians or psychologists) provide verbal information about the trial, answer questions, and obtain written informed consent from patients.

### Additional consent provisions for collection and use of participant data and biological specimens {26b}

On the consent form, participants will be informed that their data will be anonymized in case they decide to withdraw from the trial and request of their personal information to be deleted. Participants will also be asked for permission that the research team can share relevant data with people from the universities taking part in the research or from regulatory authorities where relevant. This trial does not involve collecting biological specimens for storage.

### Interventions

#### Explanation for the choice of comparators {6b}

Given the high therapeutic need and the high prevalence of ASD and the recognized urgent need for evidence-based therapy approaches, we chose to evaluate both FASTER and SCOTT&EVA against TAU within one combined pivotal study.

The two interventions FASTER and SCOTT&EVA will be compared to a TAU group. The TAU group can continue to use the usual outpatient treatments, but not a therapy that targets the core symptomatology of ASD. In Germany, “treatment as usual” is in fact rarely an ASD-specific intervention.

The SCOTT&EVA concept focuses on emotions and social interactions whereas FASTER additionally aims at areas such as stress management and a deeper understanding of social communication and interaction.

Thus, the study with two intervention groups evaluates two approaches including different aspects and range of important areas of interventions for people with ASD, both with the aim to ultimately improve social responsiveness and social competence.

#### Intervention description {11a}

After the screening phase, eligible study participants will be included into the study and assessed at baseline (T0). Following baseline measurement of six participants, all six will be assigned to one of the three groups (FASTER, SCOTT&EVA, or TAU) via cluster randomization.

The Freiburg Asperger-Specific Therapy for adults (FASTER) intervention [2, 5, 13] has been developed, validated, and published in manualized form by the Freiburg Asperger Study group since 2004 as a specific group psychotherapy concept and offers a comprehensive approach for the treatment of adult patients with high-functioning ASD, implicating good language abilities and normal or above average IQ. Weekly 120-min group psychotherapy sessions will be held for 4 months for a maximum of six participants and two therapists in a closed group. Within this timeframe, participants will receive three different therapy modules in 16 group psychotherapy sessions altogether. At the beginning of the first basis module psychoeducation and information will be given about ASD as well as rules and aims of the group therapy. The module will continue with assessing individual strengths and weaknesses of the participants, as well as stress regulation management and knowledge about autism-related and individual demands (stimulus satiation, irritation, etc.), thereby offering adapted mindfulness techniques for autism, and autism-specific strategies for self-regulation. Information about alexithymia, in-depth comparison of autistic and sociotypical rules and strategies in social interaction, non-verbal and verbal communication, as well as perspective taking will be provided in the second module. Videotaped role plays of everyday life situations (like expressing gratefulness, needs, or criticism in different types of social contacts) with individualized feedback will be the core elements in the final advanced module.

Each single group psychotherapy session comprises three different parts. A short review of last week’s outstanding events or experiences of each participant will be shared with the group for the first 20 min of the session. If participants require clarification of urgent or important group-appropriate issues, they can receive feedback from other group members. This will be followed by a short mindfulness exercise that is especially adapted to individuals with ASD. Subsequently, the actual topic of the session will be introduced.

After completion of the 4-month trial, participants will receive four monthly fresh-up group meetings in the follow-up period to keep up a therapeutic structure and motivation, to foster transfer of new knowledge and strategies into everyday life, and to allow for feedback and exchange of information. The topics of the refresher sessions comprise participants’ daily stress management, and communication issues in everyday social situations. All sessions are substituted by specific ongoing homework including self-monitoring, introspection, and analysis of social interactions that will be discussed in the next group meeting.

The Social COgnition Training Tool (SCOTT) is a computer-based manualized training program that was developed in 2008 to target social cognitive impairments in high-functioning adults with ASD. It aims to foster the recognition of 40 different emotions [20] from faces, prosody, and complex social situations, and includes more than 8000 video and audio stimuli created with 70 professional actors [21, 22]. Thereby, the SCOTT training approximates demands of real-life social contexts, displaying for example video stimuli portraying the interaction of 2–4 actors. Recently, the training has been revised and extended (SCOTT&EVA; German: Emotionen Verstehen und Ausdruecken, English: Understanding and Expressing Emotions) to include additional modules (i.e., a library of 40 different emotions) as well as an adaptive algorithm that adjusts the difficulty of the training tasks to the user’s individual performance level using a variation of the so-called Elo system (for a more detailed description, see [15]). Adapting the task difficulty according to the user’s performance can ensure a higher motivation and thus a better learning outcome. This updated version of the concept has been shown to result in high ratings on acceptance and usability measures among users [23].

The SCOTT&EVA training comprises three visually and technically appealing training modules based on diverse video and audio material:

1. Face Task: short video sequences of faces expressing emotions are displayed in three different variants of this task: video sequences of faces are either (a) simply displayed or (b) slowly revealed and the user is asked to match the faces by emotional state. A third variant of this task is that (c) the video sequences displaying faces are cut into upper and lower face parts (same identity faces) and the user is asked to match the pieces accordingly.
2. Voice Task: To improve the understanding of prosody, users are asked to match auditory vocal sequences with emotional states depicted as emotional terms.
3. Social Interaction Task: short videos (< 2 min) portraying the interaction of 2–4 actors in demanding real-life contexts are divided into sub-sequences, which are displayed simultaneously on the computer screen. The user is asked to reconstruct the correct chronology of the short movies by socio-emotional content using drag and drop functions. In a next step, the user is asked to answer questions referring to the actors’ mental states.

Furthermore, SCOTT&EVA contains a large didactic platform, detailing semantic embedding and physiological and expressional features of all emotions targeted. In this so-called Emotion Treasury module, all 40 emotions are systematically ordered and described in detail (definitions and synonyms for each emotion as well as descriptions of bodily sensations associated with and situations typically evoking those emotions). Furthermore, all emotions are depicted by face and voice stimuli.

Additionally, users receive feedback about their performance for each task and in total (Performance Scores) and time they have spent with the training (Experience Score).

The SCOTT&EVA training will be conducted by each participant individually from home via Internet. The treatment is designed to be approximately equivalent to the FASTER treatment concerning training time (a total of 32 h during the first 4 months, with a minimum training time of 2 h per week). The time every participant actually spends with SCOTT&EVA will be assessed by online tracking in SCOTT&EVA’s central database. In order to ensure correct software use and to motivate independent training at home, the participants randomized to the SCOTT&EVA group will be introduced and supervised in dealing with the computer training at the beginning of week one and ongoing once per month in the study center (with an approximate duration per supervised session of 60 min). During the follow-up phase, patients also receive one supervised fresh-up session per month (minimum duration of 30 min).

#### Criteria for discontinuing or modifying allocated interventions {11b}

Subjects are allowed to withdraw their consent to participate in the study interventions at any time without personal disadvantages and without having to give a reason. Subjects will take part in the further assessments (post-intervention, T1, and follow-up, T2), if they do not deliberately resign from the follow-up assessments.

If a subject withdraws from the trial, no additional data will be collected, but the existing data will be used for statistical analysis. If, in addition, the subject requests the collected data to be destroyed, their personal information will be erased from the database and cannot be used for further analysis.

The time of treatment or trial discontinuation and, if known, the reason for withdrawal will be documented on the CRF and in the patient file.

The investigator or Data Safety and Monitoring Board (DSMB) can also discontinue the intervention after considering the risk-to-benefit ratio, if they no longer consider further treatment of the patient according to study protocol justifiable. The date of and the primary reason for the termination and the observations available at the time of withdrawal are to be documented on the CRF. If possible, the measurements of those patients will be used. They will still be considered for the final analysis.

Drop-out patients will not be replaced.

#### Strategies to improve adherence to interventions {11c}

Rater for the screening, baseline, post-treatment, and follow-up is trained in the used tests, interviews, and computer programs: Global Assessment of Functioning (GAF), Structured Clinical Interview for DSM-5 for clinical diseases (SCID-5-CV), Autism Diagnostic Observation Scale, Version 2 (ADOS-2), Intelligence test (MWT-B and CFT 20-R), and Multifaceted Empathy Test (MET).

Therapists are trained in the application of Freiburg Asperger-Specific Therapy (FASTER) and in the administration and supervision of the Social Cognition Training Tool (SCOTT&EVA).

Video recordings from the trainings together with all manualized materials will be provided on a login-protected internet platform for raters and therapists. Thus, each study center can refresh their trainings or train new study members.

Once a month, there will be a telephone supervision for potentially emerging rating and therapy questions. Additionally, for all FASTER group therapy sessions, therapists are videotaped. These recordings will be used for supervision and adherence checks. Video cameras are placed in such a way that the study participants are not visible, only their voice is recorded. For that reason, written consent for the recording of study participants audio will be obtained.

#### Relevant concomitant care permitted or prohibited during the trial {11d}

In case of acute problems or comorbid disorders that have to be treated, all participants irrespective of their group are allowed to make use of outpatient visits, such as family doctor visits, outpatient psychiatric visits for comorbid mental disorders, doses adaptation of medication, and psychotherapy for comorbid disorders outside the study center. However, patients are not allowed to participate in specific therapy targeting the core problems of ASD during the whole trial phase and are not allowed to have received specific ASD therapy (individual and/or group therapy) for the core problems of ASD up until 6 months before inclusion into the study. It is also not permitted to change the active substance of psychotropic drugs during the study period. Participants may receive an overall maximum of up to 120 min of individual, non-ASD-specific consultation with study personnel within their period of participation.

#### Provisions for post-trial care {30}

If serious changes occur during the study period, e.g., in relation to comorbid disorders requiring treatment, these are also followed up after the study period. Appropriate measures are taken for this purpose, such as outpatient or inpatient care or referral to other specialists.

Since there are hardly any specific measures for people with ASD, participants who were randomized initially to the treatment as usual group (TAU) are offered SCOTT&EVA or FASTER after the end of the study.

### Outcomes {12}

All outcomes will be assessed at baseline (T0), post-treatment (T1, 4 months after baseline), and follow-up (T2, 8 months after baseline).

The primary endpoint of our trial is the change of the total sum score from baseline (T0) to 4 months after baseline (T1) in social responsiveness as measured with the *Social Responsiveness Scale* (SRS-A adult, parent/spouse/other, [18, 19]).

The secondary outcome measures aim to differentially assess aspects of self-reported social responsiveness, social cognition, social skills, empathy, common comorbid psychopathology, self-esteem, life satisfaction, and quality of life. Almost all questionnaires and tests (see Table 1) are well-established, widely-used instruments with excellent psychometric properties (see also the “Data collection and management” section).

The measurement for social responsiveness (parent/spouse/other) at the follow-up measurement time point (T2) is also a secondary outcome.

**Table 1:** Illustration of frequency of study visits, questionnaires, and tests

	SCR	T0	T1	T2
GAF (SCR: via telephone or personally)	x^1^	x^3^	x	x
Psychopharmacotherapy	x^1^	x	x	x
ADOS-2 (if lacking)	x^2^			
SCID-5-CV	x^2^			
CFT 20-R	x^2^			
MWT-B	x^2^			
Neo-FFI	x^2^			
AQ50	x^2^			
EQ40	x^2^			
WURS-K	x^2^			
ADHS-SB	x^2^			
TAS-26	x^2^			
Basis Documentation	x^2^			
SRS-A (patient)		x	x	x
SRS-A (parent/spouse/other)		x	x	x
SRS-A mod. (patient)			x	x
SRS-A mod. (parent/spouse/other)			x	x
MET (computer test)		x	x	x
BDI-II		x	x	x
SASKO		x	x	x
MSWS		x	x	x
FLZ^M^		x	x	x
WHOQoL-Bref		x	x	x
Freiburg Event List			x	x
Partnership Questionnaire (if appropriate)		x	x	x

*Abbreviations: GAF*, Global Assessment of Functioning (assessed by a telephone interview); *ADOS-2*, Autism Diagnostic Observation Scale; *SCID-5-CV*, Structured Clinical Interview for DSM 5, Disorders, Clinician Version; *BDI-II*, Beck Depression Inventory; *CFT 20-R*, Culture Fair Intelligence Test Revised; *MWTB*, Mehrfachwahl-Wortschatz-Intelligenz-Test; *AQ50*, Autism Spectrum Quotient with 50 items; *EQ40*, Cambridge Behaviour Scale with 40 items; *WURS-K*, Wender Utah Rating Scale, short form; *ADHS-SB*, Attention Deficit Hyperactivity Disorder, self-report; *TAS-26*, Toronto Alexithymia Scale; *SRS-A*, Social Responsiveness Scale (self-report, patient); *SRS-A (parent/spouse/other)*, Social Responsiveness Scale from parent or spouse, external assessment; *SRS-A mod. (patient; parents/spouse/other)*, modified version of SRS-A for measuring differences from baseline (T0) to post-treatment assessment (T1) and post-treatment assessment (T1) to follow-up (T2) in five gradations (considerably aggravated to considerably improved); *Neo-FFI*, Neo Five-Factor-Inventory; *MET*, Multifaceted Empathy Test; *FLZ*^*M*^, Life Satisfaction Questionnaire; *MSWS*, Multidimensional Self-worth Scale; *WHOQOL*, WHO Quality of Life; *SASKO*, questionnaire for social anxiety and social skills deficits; *Freiburg Event List*, questionnaire about life events; Partnership questionnaire for partner of the participant, if living in partnership and self-report; *SCR*, screening; assessment—*x1* first screening assessment (SCR I); *x2* second screening assessment (SCR II); *x3* have to be measured again if the last measurement was more than a month ago

#### Primary outcome

For social responsiveness, the *SRS-A* [19, 24] is used as an external assessment instrument that had to be completed in by caregivers, spouses, or other people who have sufficient insight into the respective participant’s social communication and interaction. The SRS-A is an internationally widely-used, well-established instrument with a patient and a caregiver/spouse version with very good sensitivity. The German adult version has been validated [18]. Due to psychometric properties and since it provides internationally comparable results, it is a good instrument to assess changes in social responsiveness as a consequence of therapy [24–27]. Good reliability and validity of the German adult version have been shown (Cronbach’s alpha .89) [24].

The SRS-A cut-off score for adults is 67 with a sensitivity of .85, and a specificity of .83 for autism spectrum disorder in comparison to patients with other mental disorders and typical developed individuals. There is no normed German version of SRS-A available so far, but a validation has already been published [24].

#### Secondary outcomes

Additional SRS-A measurements will be done for our secondary objectives.

The *Social Responsiveness Scale for adults*, second edition (SRS-A, 19), is also available in the form of a self-report. Compared to the SRS-1, the SRS-A has the additional variant for self-assessment and is eligible from 18 years onwards. The 65 items of the SRS-A are almost identical to the SRS-1 in terms of content, but in the SRS-A self-assessment, the statements are given in an ego perspective. The questionnaire comprises five subscales: social consciousness, social cognition, social communication, social motivation, and autistic mannerism. The Cronbach’s alpha for the total score is .89 for individuals within the autism spectrum.

As secondary outcome, we will assess changes in social responsiveness via the SRS-A self-report [24–26] for the global sum score as well as for each of the five subscales. With a modified answer version of the SRS-A, we will additionally ask about the specific difference between the beginning and the end of treatment with a five point Likert scale between “much worse,” “somewhat worse,” “stayed the same,” “somewhat better,” and “much better” instead of the original scale. The modified version is used for both the external evaluation and the self-report.

Furthermore, we apply the Global Assessment of Functioning (GAF), which is part of the Structured Clinical Interview for DSM-IV [17] and frequently used in clinical practice.

Social cognition is tested via the computer-based *Multifaceted Empathy Test* (MET, [28]), which is an instrument for measuring both cognitive and emotional empathy. The MET consists of 26 pictures of emotional scenes and participants are asked to rate the emotional state of the person shown in the picture out of four possible answers (cognitive empathy) and to rate how strongly they feel the emotion of the person in the picture using a 9-point Likert-scale (emotional empathy).

The MET is an objective photo-based test that has specifically been designed and validated in adults with ASD [28]. It allows the calculation of a performance score for correct mental state inference and a valid estimate of the level of emotional engagement/responsiveness towards others.

Comorbid disorders like depression and social anxiety are measured. Depression will be measured with the *Beck Depression Inventory*, second edition (BDI-II, [29]). It is a widely used questionnaire for depression (Cronbach’s alpha = .92). Social anxiety is measured with the *Questionnaire for Social Anxiety and Social Skills Deficits* (SASKO, [30]). It has five different subscales (anxiety for speaking and take center stage, anxiety for rejection, deficit in interaction, deficit in information processing, and loneliness). The first four dimensions build the anxiety deficit structure of the questionnaire. The sum score of these four dimensions with 40 items have good internal consistency (Cronbach’s alpha between .92 and .94 from four different studies) [30].

Self-worth is measured with the *Multidimensional Self-worth Scale* (MSWS, [31]). It has six subscales (emotional self-worth, social self-worth – safety in contact, social self-worth – handling of criticism, performance-related self-worth, self-worth in physical attractiveness, self-worth in sportiness), resulting in two higher-order dimensions (general self-worth, body related self-worth), and overall self-worth as total sum score. Internal consistency for the subscales is between .75 and .87, the higher-order dimensions between .85 and .92 and the overall score has a Cronbach’s Alpha of .93.

Life satisfaction is measured with the *Life Satisfaction Questionnaire* (FLZ, [32]). It consists of a general part and a part focusing on physical healthiness. In the study, the general part of the questionnaire is used with eight questions for different areas of life. All questions are answered in relation to importance and satisfaction. The scale about the importance of life areas has a Cronbach’s alpha of .82.

Quality of life is measured with the *Quality of Life Questionnaire from the WHO* (WHOQoL-BREF, [33]). It consists of four scales: physical and psychological health, social relationship, and environment. For the short version of the questionnaire used here, the internal consistency lies between .66 and .80 for the four scales, and between .83 and .84 for the total score.

Two other questionnaires have been constructed in Freiburg especially for adults with autism and are not validated until now. We use specifically formulated questions for potentially occurring life events and a partnership questionnaire where either partners or only one may be autistic. To date, no comparable questionnaires are available in Germany.

The *Freiburg Event List* (FEL) will be used to assess non-treatment life events and is included to evaluate possible moderation effects at post-treatment and follow-up.

#### Demographic and baseline information

During screening, different measures are made in order to get important initial values for the description of the study participants. Autistic symptoms are measured via self-report with the *Autism Spectrum Quotient* (AQ, [34]) and the *Empathy Quotient* (EQ, [35]). The authors of the AQ state that 80% of people within the autism spectrum obtain values of ≥32, while the values for non-autistic persons are < 32. The 50 items include questions on social skills, attention shifting, communication, attention to detail, and imagination. It is a questionnaire for self-report and is suitable for adults. The Cronbach’s alpha of the AQ for the overall sum score is .79. The EQ is used to differentiate between persons with ASD and non-autistic persons with regard to the dimension empathizing. The screening questionnaire comprises 40 items from which the EQ is calculated. Most non-autistic persons reach values of ≥30 whereas 80% of persons with ASD reach values < 30. The questionnaire is used for adults. The Cronbach’s alpha for the sum score is .92.

Alexithymia will be measured with the German version of the *Toronto-Alexithymia-Scale-26* (TAS -[25, 36]). It is used to measure emotional blindness (alexithymia) and is composed of three subscales: “Difficulties in identifying feelings,” “Difficulties in describing feelings,” and “Externally oriented thinking style.” An overall alexithymia score can be calculated from the three scales. The TAS-26 consists of 26 items and the overall scale has a Cronbach’s alpha of .81.

ADHD symptoms are assessed with the *Wender-Utah Rating Scale* (WURS-K, [37]) for symptoms in childhood (Cronbach’s alpha of .91) and with the *ADHD self-report* (ADHD, self-report, [38]) for current symptoms (Cronbach’s alpha between .70 and .90).

Intelligence will be measured with a culture fair test and the multiple choice vocabulary intelligence test. The *Culture Fair Intelligence Test*, second edition (CFT 20-R, [39]), measures the general intellectual level (basic intelligence) in terms of the “General Fluid Ability” according to Cattell (1963). This concept can be described as the ability to recognize figural relationships and formal-logical thinking problems with varying degrees of complexity and to process them within a certain time. The test is based on language-free and descriptive test tasks. The CFT 20-R consists of two similarly structured test parts, each with four subtests (series continuation, classifications, matrices and topological conclusions). The number of items in part 1 of the revised version was increased from 46 items to 57 items and was used to determine the IQ. The Cronbach’s alpha is .95.

The *Multiple choice vocabulary intelligence test* (German: Mehrfachwahl-Wortschatz Intelligenztest, MWT-B, [40]) is a very efficient test for measuring general intelligence levels that is frequently used in practice. The test mainly measures crystalline intelligence. It consists of 37 items, which are arranged in ascending order of difficulty.

The *NEO-Five-Factors-Inventory* (NEO-FFI, [41]) will be used to assess general personality traits by self-report. As the DSM personality interviews such as the Structured Clinical Interview for DSM-5 (SCID-5-PD, [42]) may lead to multiple diagnoses of personality disorders related to the high overlap of ASD symptoms, we considered a more general description of the personality to be more appropriate. The five factors of the personality questionnaire comprise neuroticism, extraversion, openness, conscientiousness, and agreeableness. Internal consistency (Cronbach’s alpha) of the five factors lies between .72 and .87.

The following *additional information about the participants* is collected: age, gender, native speaker, marital status, age and sex of siblings and own children, current life situation, school leaving certificate, education, profession, family members with ASD diagnosis, possible addiction-related Internet use in various areas, current psychiatric and psychotherapeutic treatments, time point and diagnostic center at ASD diagnosis, and years of therapeutic ASD treatment.

### Participant timeline {13}

The measurement points are displayed in Table 2.

**Table 2:** T_F/S_ (treatment) = FASTER: 16 sessions at 120 min, group psychotherapy, 1/week and a single treatment at beginning and end of treatment; SCOTT&EVA: 32-h computer training with a minimum of 2 h/week, this will be controlled by online tracking and supervised training at the beginning of week one and ongoing once per month; B_F/S_ = Booster sessions FASTER: 4 sessions at 120 min 1/month; SCOTT&EVA: 4 sessions at 30 min 1/month. Screening-visits –t_2_ and –t_1_, t0, t1, t2 are measurements for all groups FASTER, SCOTT&EVA, and TAU

	Study period							
	Enrolment		Allocation					
Visit No./timepoint	SCR I −t_**2**_	SCR II −t_**1**_	t0		FASTER or SCOTT&EVA	t1		t2
Procedure	Screening				Treatment phase		Follow-up phase	
**Month**	**−2**	**−1**	**0**	**Randomization**	**1–4**	**4**	**5–8**	**8**
**Inclusion/exclusion criteria 1**	●							
**Inclusion/exclusion criteria 2**		●						
**Written informed consent**		●						
**Baseline (T0)**			●					
**Interventions**					T_F/S_			
**Post-treatment measurement (T1)**						●		
**Booster Sessions for treatments**							B_F/S_	
**Follow-up measurement (T2)**								●
**AE/SAE**			●		●	●	●	●
**Psychopharmacotherapy**	●	●	●		●	●	●	●

### Sample size {14}

The sample size calculation is based on the primary outcome measure “mean difference of the SRS-A score between baseline and 4 months after baseline”. The aim is to show that at least one of the two treatment protocols (SCOTT&EVA or FASTER) is superior to TAU which results in a union-intersection test. The sample size calculation is based on an ANCOVA model adjusted for the baseline SRS-A score. Since both models are well comparable, the power of the finally applied mixed model repeated measures (MMRM) is expected to be close to the intended value used for sample size calculation based on an ANCOVA. To obtain an estimate for the correlation between baseline and 4-months value, we analyzed internal data of 17 patients with a time difference of 9 months between measurements of SRS-A score. This yielded a correlation coefficient of .27 (95% CI [−.24; .66]). Hence, we use a conservative correlation of .2 for sample size calculation. The standardized treatment effects for SCOTT&EVA versus TAU and FASTER versus TAU are both assumed to be equal to 0.4. These prior assumptions are based on previous research results [43].

The global two-sided significance level is 0.05. The two test statistics referring to the comparison of TAU versus SCOTT&EVA and TAU versus FASTER are correlated due to the common comparator TAU. This correlation is determined by the ratio of variances of the group effect estimators. We assume equal variances in TAU and SCOTT&EVA and a small intra-group correlation of 0.05 in FASTER. Since SCOTT&EVA and TAU are no group therapies, we expect, if at all, a very small intra-group correlation caused by cluster randomization. Conservatively, we assume an intra-group correlation of 0.01 in these treatments. This results in a design effect of 1.25 inflating the variance in FASTER and a design effect of 1.05 inflating the variances in SCOTT&EVA and TAU. Hence, the correlation of test statistics is given by $\frac{1}{\sqrt{\left(1.05+1.05\right)\bullet \left(1.05+1.25\right)}}=0.455$. The correlation-adjusted local significance levels are computed from a bivariate normal distribution and are given by 0.026632 [44]. Applying these local levels guarantees that the family-wise error rate is controlled by the global two-sided significance level of 0.05.

The required sample size to find a significant effect in at least one of the two comparisons with a power of 0.8 under the above-described assumptions and at correlation-adjusted local levels of 0.026632 is given by 87 patients per group. Based on experiences in our previous FASTER and SCOTT&EVA trials, drop-out rates of about 20–25% are expected [13, 21]. Thus, we conservatively estimate the drop-out rate for the current study by 25% which compares well to similar interventions in adult ASD [43, 45]. To obtain a sample size that can be divided by the cluster size of 6, 120 patients for each group will be required to be randomized resulting in a total sample size of 360.

### Recruitment {15}

Participant recruitment is expected to begin in March 2021 and will continue for 28 months.

Each of the participating centers has consented to include a certain number of patients according to their usual quantities of diagnostics and treatment of autistic patients. The study centers keep waitlists for diagnostics and treatment and will also use existing patient files for screening. Flyers with cover letters for medical practices and autism centers as well as press information have been prepared with general information about the study. These materials can be adapted with each study center’s contact information and distributed within their local networks. Some of the study centers have well-established web pages in place to advertise clinical trials especially for their autistic patients. In addition, Germany’s largest society for autistic people and their caregivers, Autismus Deutschland e.V., has been requested to include information about the study on their webpage for advertising trials looking for autistic participants in the German speaking area.

## Assignment of interventions: allocation

### Sequence generation {16a}

Subjects are randomized by cluster randomization in an allocation ratio of 1:1:1. The cluster size is set to 6 patients per cluster. This means that groups of 6 participants are built within each center, and these groups are randomly assigned to one of the three study arms. The randomization will not be stratified by center or other factors. An Internet-based randomization system (http://www.randomizer.at) will be used and the allocation sequence is created by computer-generated random numbers.

All transactions will be logged. The software’s GCP-compliance has been confirmed by the Austrian Federal Office for Safety in Health Care (BASG). Details of the randomization scheme will be specified in an external document accessible to the Institute of Medical Biometry and Informatics (IMBI, Heidelberg) only to minimize selection bias.

### Concealment mechanism {16b}

The allocation sequence is implemented and concealed by the randomization tool “randomizer” (http://www.randomizer.at). Study staff which do not have to be blind will insert always six in the study included study participants’ identification codes into the randomizer online. All already randomized groups can be viewed by the study staff of the corresponding center and from the IMBI. Subjects withdrawn from the trial will retain their identification codes (e.g., randomization number, if already given). New subjects must always be allotted a new identification code.

### Implementation {16c}

The study-specific randomization tool of http://www.randomizer.at will be set up by the Institute of Medical Biometry and Informatics (IMBI, Heidelberg). All study participants who give written consent and meet the eligibility criteria will be randomized in clusters. Raters from each center decide on the inclusion of potential study participants based on clearly defined criteria. Randomization will be requested from the rater of each study center via the online randomization tool “randomizer.at” from the University of Graz, Austria. Only the data manager of the Institute of Medical Biometry and Informatics (IMBI, Heidelberg) has complete access to the stratification and randomization process. All centers add six new participant identification numbers into the randomizer for a complete cluster and will then receive the random allocation to one of the three groups (group therapy FASTER, web-based training SCOTT&EVA or TAU).

## Assignment of interventions: blinding

### Who will be blinded {17a}

Randomization takes place after baseline. Study staff from each study center who measure post-treatment (T1) and follow-up (T2) are blinded regarding group assignment. Rater and therapy rooms are far apart, to support the blinding of the raters throughout the whole course of the study. Raters log at the measurement time points T1 and T2 for each study participant whether they are blinded or not. Due to study design and significantly different interventions in the study arms, therapists, study participants, and additional persons who are responsible for the external assessment for social responsiveness are not blinded. The biometrician and his representative will remain blinded until data base closure.

### Procedure for unblinding if needed {17b}

No procedure for unblinding of therapists is necessary. Therapists always have information about the allocation of each study participant. If unblinding of raters takes place by accident, the unblinding is recorded and another rater will perform the future measurements (T1 or T2).

## Data collection and management

### Plans for assessment and collection of outcomes {18a}

A detailed description of the study instruments and their validity can be found in the section “Outcomes”.

Raters are trained for 2 days in the Structured Clinical Interview for DSM-5 for clinical diseases (SCID-5-CV), the Autism Diagnostic Observation Scale (ADOS-2), the Global Assessment of Functioning (GAF), the Multifaceted Empathy Test (MET, computer test) and IQ testing for the Culture Fair Intelligence Test (CFT 20-R) and the complete process of data acquisition.

Additionally, all trained procedures are video recorded and are available via Internet access for all raters. Also available are frequently asked questions about the procedures via Internet access. Monthly supervisions via video conference will be held for best knowledge approximation between centers.

If new raters have to be trained, they will use the video-recorded information about training and take also part in the monthly supervisions via video-conference.

Therapists for FASTER and SCOTT&EVA are trained for 2 days regarding all sessions and procedures. Additionally, all trained procedures are video-recorded and are available for all therapists via Internet access. Frequently asked questions about the procedures are also available via Internet access. Monthly supervisions via video conference are offered for best knowledge approximation between centers.

If new therapists have to be trained, they use the video-recorded information about training and take also part in the monthly supervisions via video conference.

The training measures for raters and therapists of the study are documented in training logs. Raters and therapists can only be accepted into the study team after successful completion of the training. The recording must be authorized by the center’s principal auditor. The training logs are checked via the monitoring visits.

Data collection forms for each center can be found in the investigator site file (ISF) as well as in an internet-based platform that is accessible to all raters and therapists. In addition, frequently asked questions (FAQ) about study contents are available for all participating members of the study centers.

### Plans to promote participant retention and complete follow-up {18b}

Between T1 and T2, both FASTER and SCOTT&EVA participants are offered monthly refresher sessions in the respective study center. The refresher sessions are implemented to support the transfer of skills and knowledge gained during the intervention phase into daily life.

TAU patients are offered a compensatory FASTER or SCOTT&EVA program after completion of their trial participation. SCOTT&EVA participants will also be offered an additional FASTER group therapy after completion of their trial participation if requested. Currently, there are few treatment options for the study participant group, so participation in this study is most likely motivating. In case of protocol violations or withdrawals, the subjects will still take part in the further assessments if they do not deliberately resign also from the follow-up assessments.

### Data management {19}

Case report forms (CRFs) are paper-based. Source data will be collected in each center from the rating and therapy staff and will be transferred into the CRFs. Source data is stored in the respective center. Source data and transmission to the CRFs will be checked via monitoring by on-site visits in each study center. A copy of each CRF will be made and the original will be sent to the Institute of Medical Biometry and Informatics (IMBI, Heidelberg).

The Institute of Medical Biometry and Informatics (IMBI, University Hospital Heidelberg) is responsible for the data management within the trial. In order to ensure that the database reproduces the case report forms (CRF) correctly, the IMBI accomplishes a double entry of data. In order to guarantee high quality of data, the completeness, validity, and plausibility of data as defined in a data validation plan will be checked using validating programs, which will generate queries. A tracking system for CRF data and queries will be established to guarantee that data is managed in a timely manner. The investigator or the designated representatives are obliged to clarify or explain the queries. If no further corrections are to be made in the database it will be closed and used for statistical analysis. All data management procedures will be carried out on validated systems and according to the current Standard Operating Procedures (SOP) of the IMBI that guarantee an efficient conduct which is in compliance with GCP.

At the end of the study, the data will be transformed into different data formats (e.g., csv-files) to ensure that it will be possible to reuse it. The principle investigator will retain the originals of all CRFs and the trial data for long-term preservation. After completion of the study, we plan to make the publication data and the primary data publicly available for re- and meta-analyses.

### Confidentiality {27}

The data obtained in the course of the trial will be treated pursuant to the Federal Data Protection Law (Bundesdatenschutzgesetz, BDSG).

During the clinical trial, subjects will be identified solely by means of their individual identification code (screening number, randomization number). Trial findings stored on a computer will be stored in accordance with local data protection law and will be handled in strictest confidence. Distribution of these data to unauthorized persons is strictly prevented. The appropriate regulations of local data legislation will be fulfilled in its entirety.

The subject consents in writing to release the investigator from his/her professional discretion in so far as to allow inspection of original data for monitoring purposes by authorized persons (monitors, auditors). Authorized persons (clinical monitors, auditors) may inspect the subject-related data collected during the trial ensuring the applicable data protection law.

The investigator will maintain a subject identification list (subject numbers with the corresponding subject names) to enable records to be identified. Subjects who did not consent to circulating their pseudonymized data will not be included into the trial.

This protocol, the CRFs, and other trial-related documents and material will be handled with strictly confidentiality and will not be disclosed to third parties except with the express prior consent of the Lead Investigator. Staff of the investigators involved in this study are also bound by this agreement.

### Plans for collection, laboratory evaluation, and storage of biological specimens for genetic or molecular analysis in this trial/future use {33}

There will be no biological specimens collected (see the “Additional consent provisions for collection and use of participant data and biological specimens {26b}” section above).

## Statistical methods

### Statistical methods for primary and secondary outcomes {20a}

#### Trial populations to be analyzed

The primary analysis will be performed for the full analysis set which comprises all patients randomized into the trial. In this set, every patient is analyzed according to the randomized group.

The per-protocol set will comprise all patients who were treated according to the randomized treatment without further major protocol violations.

The safety set will comprise all patients of the full analysis set and will allocate the patients to the treatment they actually received, regardless of randomization.

The allocation of each patient to the different study populations will be defined and explained in further detail in the statistical analysis plan (SAP).

#### Estimands

In the recently released ICH E9 (R1) addendum on estimands and sensitivity analysis in clinical trials, the estimands framework is recommended as clear and transparent definition of “a structured framework to strengthen the dialogue between disciplines involved in the formulation of clinical trial objectives, design, conduct, analysis and interpretation, as well as between sponsor and regulator regarding the treatment effect(s) of interest that a clinical trial should address” [46]. Such an estimand can be defined through the population of interest, variable of interest, specification of how intercurrent events are handled, and summary measure. The specification of how intercurrent events are handled is referred to as intervention effect in the following. This way, a more precise definition of the treatment effect of interest in relation to the study objective(s) is enabled. Based on such an estimand, adequate methods to estimate this estimand can be chosen. In the following, the primary estimand corresponding to the primary objective is described. If unforeseen intercurrent events will occur frequently during the course of the trial, they will be specified in the statistical analysis plan. By only reporting pooled data without information on the treatment group allocation to the supervising and the study biometrician until the database is locked, blinding will be maintained. In the statistical analysis plan, further sensitivity estimands will also be described.

#### Primary estimand

##### Population

The population is defined through appropriate inclusion/exclusion criteria to reflect the targeted patient population.

##### Variable

The variable is the SRS-A score (parent/spouse/other).

##### Intervention effect

Possible intercurrent events and the strategies to handle them are as follows. Serious adverse events and adverse events will be ignored for the primary analysis (treatment policy strategy). The same strategy will be applied for all other intercurrent events as, e.g., treatment withdrawal or unforeseen in-patient stay.

##### Summary measure

The summary measure is the difference in the SRS-A (parent/spouse/other) between 4 months after baseline (T1) and the baseline value (T0). The applied test is described in the next subsection.

#### Analysis

Let μ denote the unknown true mean difference of the SRS-A score between baseline and 4 months after baseline. The following two primary local test problems are assessed:

$$ {\mathrm{H}}_{0,\mathrm{SCOTT}\&\mathrm{EVA}}:{\upmu}_{\mathrm{SCOTT}\&\mathrm{EVA}}={\upmu}_{\mathrm{TAU}}\ \mathrm{versus}\ {\mathrm{H}}_1:{\upmu}_{\mathrm{SCOTT}\&\mathrm{EVA}}\ne {\upmu}_{\mathrm{TAU}} $$

and

$$ {\mathrm{H}}_{0,\mathrm{FASTER}}:{\upmu}_{\mathrm{FASTER}}={\upmu}_{\mathrm{TAU}}\ \mathrm{versus}\ {\mathrm{H}}_1:{\upmu}_{\mathrm{FASTER}}\ne {\upmu}_{\mathrm{TAU}} $$

The aim is to show that at least one of the two local alternative hypotheses holds true. For each pairwise comparison (TAU versus FASTER and TAU versus SCOTT&EVA) a Mixed Model Repeated Measures (MMRM) will be applied. It includes the difference of the SRS-A score between baseline and 4 months after baseline as the dependent variable, the baseline SRS-A score, and the group allocation as fixed effects, and the center and the cluster as random effects. The MMRM uses all available changes from baseline SRS-A score for all patients for model estimation. In the “Sample size {14}” section, the local significance level of .026632 has been deduced. In case that at least one of the null hypotheses specified above can be rejected at the local significance levels of .026632, we hierarchically assess additionally the following test problem:

$$ {\mathrm{H}}_{0,\mathrm{SCOFA}}:{\upmu}_{\mathrm{SCOTT}\&\mathrm{EVA}}={\upmu}_{\mathrm{FASTER}}\ \mathrm{versus}\ {\mathrm{H}}_1:{\upmu}_{\mathrm{SCOTT}\&\mathrm{EVA}}\ne {\upmu}_{\mathrm{FASTER}} $$

at the full two-sided level of 0.05 in a confirmatory manner. In case neither H0, SCOTT&EVA nor H0, FASTER can be rejected, the comparison between the FASTER and the SCOTT&EVA arm is done descriptively. The confirmatory analysis of the primary efficacy endpoint corresponds to the primary estimand and will be conducted in the full analysis set.

As a sensitivity analysis to the primary efficacy analysis, corresponding MMRMs with additional covariates/factors given by IQ, age, gender, ASD symptom severity, MET test result, and center will be conducted. Furthermore, the separate items of the SRS-A score are analyzed individually to investigate which items differ between the treatment groups. Details of these analyses will be specified in the statistical analysis plan (SAP).

Exploratory analyses will investigate moderation of treatment effects by conducting the primary MMRM considering the following additional variables: IQ, age, gender, center, years of therapy experience with autism, depressiveness (BDI), self-confidence (MSWS), social competence (SASKO), and life events (FEL). To optimize treatment selection for individual patients, these covariates will be used to estimate individualized treatment effects from parameter estimates to construct optimal individualized treatment rules and to clarify for whom a treatment works best [47].

Descriptive methods will be used for the analysis of the secondary outcomes, including the calculation of appropriate summary measures of the empirical distribution as mean, median, standard deviation, 1st and 3rd quantile for continuous outcomes, and absolute and relative frequencies for count data. Furthermore, 95% confidence intervals and descriptive two-sided *p*-values will be reported. Graphical methods will be applied to visualize the findings. The safety analysis includes calculation of frequencies and rates of adverse and serious adverse events. Additionally, as supplementary analysis the primary endpoint will be evaluated in the per-protocol population. Furthermore, statistical methods are used to assess the quality of data. All analyses will be done using Statistical Analysis Software (SAS) version 9.4 or higher.

Analyses will be defined in detail in the SAP which has to be authorized before data base closure by the two involved biometricians and the lead investigator.

### Interim analyses {21b}

No interim analysis is planned.

### Methods for additional analyses (e.g., subgroup analyses) {20b}

Subgroup analyses will be performed regarding the indication (F84.0, F84.5, F84.1 if DSM criterion A, social communication and social interaction is true).

### Methods in analysis to handle protocol non-adherence and any statistical methods to handle missing data {20c}

The trial populations including the per protocol analysis set are defined in the section “Statistical methods”.

The MMRM applied for the analysis of the primary endpoint takes into account missing data. It uses all available changes from baseline SRS-A score for all patients for model estimation. If a patient has a missing change from baseline SRS-A score, the model assumes that the patient’s missing SRS-A score is comparable to the observed SRS-A score of another patient having similar baseline characteristics and a comparable course of change from baseline.

### Plans to give access to the full protocol, participant-level data, and statistical code {31c}

After completion of the trial, the data obtained by the study will be summarized and analyzed according to the protocol and hereafter published in a peer-reviewed journal. This will include an appendix with the full study protocol. Requests for data sharing will be reviewed on an individual basis by the Steering Committee. The data sharing process will comply with the good practice principles for sharing individual participant data from publicly funded clinical trials, and data sharing will be undertaken in accordance with the required regulatory requirements. Especially, the privacy of the patient data (i.e., sharing of pseudonymized data only) will be followed throughout the entire study.

## Oversight and monitoring

### Composition of the coordinating center and trial steering committee {5d}

*Lead Investigator*

Prof. Dr. Ludger Tebartz van Elst

Medical Center – University of Freiburg

Department of Psychiatry and Psychotherapy

Hauptstrasse 5, 79104 Freiburg, Germany

*Biometrician and Data Management*

Institute of Medical Biometry and Informatics, University Hospital Heidelberg

Prof. Dr. Meinhard Kieser

Marsilius-Arkaden, Turm West,

Im Neuenheimer Feld 130.3

69120 Heidelberg, Germany

*Project Manager*

Medical Center – University of Freiburg

Department of Psychiatry and Psychotherapy

Dr. Thomas Fangmeier

Hauptstrasse 5

79104 Freiburg, Germany

*Monitoring*

Coordination Center for Clinical Trials (KKS), University Hospital Heidelberg

Dr. Steffen P. Luntz

Marsilius-Arkaden - Turm West

Im Neuenheimer Feld 130.3

69120 Heidelberg, Germany

*Lead Site Investigators*

Berlin:

Prof. Dr. Isabel Dziobek

Berlin School of Mind and Brain, Department of Psychology

Faculty of Life Sciences, Humboldt-Universitaet

Unter den Linden 6, 10099 Berlin, Germany

Dresden:

Prof. Dr. Veit Roessner

Faculty of Medicine, TU Dresden

Department of Child and Adolescent Psychiatry and Psychotherapy

Fetscherstraße 74, 01307 Dresden, Germany

Essen:

Prof. Dr. med. Katja Koelkebeck

LVR-Hospital Essen, Department of Psychiatry and Psychotherapy, Medical Faculty, University of Duisburg-Essen,

Virchowstr. 174, 45147 Essen, Germany

Mannheim:

Dr. Oliver Hennig

Central Institute for Mental Health

J 5, 68159 Mannheim, Germany

Tübingen:

Prof. Dr. Dr. Dirk Wildgruber

Department of Psychiatry and Psychotherapy

University of Tuebingen

Calwerstr. 14, 72076 Tuebingen, Germany

*Steering Committee*

Prof. Dr. L. Tebartz van Elst, Prof. Dr. I. Dziobek, Dr. T. Fangmeier, Prof. Dr. M. Kieser

### Composition of the data monitoring committee, its role and reporting structure {21a}

The independent Data Safety and Monitoring Committee (DSMC) will consist of two medical scientists and one statistician with longstanding experience in clinical trials: Prof. Dr. Sven Bölte (Center of Neurodevelopmental Disorders, Department of Women’s and Children’s Health, Karolinska Institutet, Stockholm), Prof. Dr. Konrad (Clinic for Paediatric Psychiatry, Psychosomatic Disorders and Psychotherapy, University Medical Center, RWTH Aachen), and Prof. Dr. Martin Hellmich (Institute of Medical Statistics and Computational Biology, University of Cologne). The function of the DSMC is to monitor the course of the study and, if necessary, to give a recommendation to the coordinating investigator regarding discontinuation, modification or continuation of the study. The underlying principles for the DSMC are ethical and safety aspects for the patients. The DSMC is responsible to examine whether the conduct of the study is still ethically justifiable, whether security of the patients is ensured and whether the process of the study is acceptable. The DSMC will be informed about adherence to the protocol, patient recruitment, and observed serious adverse events. The DSMC will receive the corresponding reports at regular intervals (every 6 months). Recommendations on the further continuation or modification of the study will be given to the coordinating investigator. The composition and responsibilities of the DSMC, the structure and procedures of its meetings, and its relationship to other study team members will be documented in a separate DSMC charter.

### Adverse event reporting and harms {22}

Following ICH-GCP, the definition of an adverse event (AE) is adapted as follows: an AE is any untoward occurrence in a subject participating in a study, which does not necessarily have a causal relationship with the study treatment. An AE can therefore be any unfavorable and unintended sign, symptom, or disease temporally occurring alongside with the treatment (FASTER/SCOTT&EVA/TAU), whether or not related to the treatment.

The following will be defined as adverse events for the treatment study FASTER/SCOTT&EVA:

1. Increase of comorbid symptoms, e.g., substantial depression symptoms from none or mild to moderate symptoms ≥19 (BDI-II) or a significant worsening from moderate to severe symptoms (≥ 30); severe obsessive-compulsive or anxiety disorder; constant and excessive interactional problems (dispute with group members) or sensory overflow that prevent participation in group therapy.
2. Significant recurrence of comorbid disorders.
3. Appearance of new comorbid symptoms/disorders as described in 1 or new diagnosis.
4. Occurrence of new passive suicidal thoughts (suicide questionnaire, item 4 and 5 positive).
5. Occurrence of active suicidal thoughts or plans (suicide questionnaire, item 6 or higher positive).
6. Changes in medication class (not dose adjustment) of antidepressants, benzodiazepines, typical or atypical neuroleptics, stimulants, and mood stabilizers.
7. Occurrence of constant and excessive problems in the relationship between patient and therapist.
8. Private burdens like divorce, death of a family member, and relocation.
9. Occupational burdens like job change, loss of employment, or significant changes in frame conditions of existing employment.
10. Vocational reintegration burdens (measures by the employment office; rehabilitation measures, which lead to conflicts of dates with the study intervention).

A pre-existing disorder or symptom will not be considered an adverse event unless there will be an untoward change in its intensity, frequency or quality. This change will be documented by an investigator.

Surgical procedures themselves are not AEs; they are therapeutic measures for conditions that require surgery. The condition for which the surgery is required may be an AE. Planned surgical measures permitted by the clinical trial protocol and the condition(s) leading to these measures are not AEs if the condition leading to the measure was present prior to inclusion into the trial.

AEs are classified as “non-serious” or “serious.”

### Frequency and plans for auditing trial conduct {23}

Monitoring will be done by on-site visits and frequent communication (letters, telephone, fax, e-mail) by a clinical monitor according to SOPs of the KKS. The monitor will ensure that the trial is conducted according to the protocol and regulatory requirements by review of source documents, entries into the CRFs and essential documents. Therefore, the investigator will allow the monitor to verify these documents and will provide support to the monitor at all times. The monitor will document the visits in a report for the sponsor. The site will be provided with a follow-up letter of the findings and the necessary actions to be taken.

In total, on average one to two monitoring visits are carried out per center and year, depending on the number of study participants included. The monitoring is carried out by the Coordination Center for Clinical Trials in Heidelberg (KKS).

As the monitoring strategy will consider current aspects of risk-based quality management, frequency of monitoring activities per site will vary depending on recruitment, experience, and general performance, e.g., quality of documentation of the individual trial centers. Details of monitoring will be defined in the monitoring plan.

If there are major findings during monitoring or an audit, the investigational site might be closed by the Lead Investigator.

### Plans for communicating important protocol amendments to relevant parties (e.g., trial participants, ethical committees) {25}

Any modifications to the protocol which may have an impact on the study, potential benefit for the patient or may affect patient safety, including changes of study objectives, study design, patient population, sample sizes, study procedures, or significant administrative aspects, will require a formal amendment to the protocol. This will be decided jointly with the Steering Committee, the study centers, and will undergo approval by the Ethics Committee.

Administrative changes of the protocol are minor corrections and/or clarifications that have no effect on the way the study is conducted. These administrative changes will be approved within the Steering Committee and the study centers.

## Dissemination plans {31a}

The primary outcome publications of the study will present outcome data regarding social responsiveness between baseline and post-treatment, as well as secondary outcomes for social responsiveness, social cognition, psychological well-being, quality of life, self-worth, and life satisfaction for all three measurement points.

In addition, outcomes will be presented on congresses, symposia, workshops, etc., if applicable.

## Discussion

The purpose of this study is to validate existing data for the two different treatment concepts FASTER and SCOTT&EVA in a multi-center, randomized phase-III trial. FASTER and SCOTT&EVA concepts are among the most elaborated German treatment concepts for adults with ASD in line with modern therapy standards and have demonstrated feasibility and yielded good phase-II data. The study protocol meets the essential standards of modern research in psychotherapy. Since the required sample size to find a significant effect with a power of 0.8 is given by 87 patients per group, a number of 120 participants per group and a total sample size of 360 participants will be sufficient to find an effect (assuming there is one). In order to minimize the differences among groups, the participants will be randomized to treatment and control conditions (randomized controlled trial). The use of standardized diagnostic instruments for the assessment of eligibility as well as primary and secondary efficacy analysis facilitates ecologically valid outcomes. A defined primary endpoint before trial initiation and a registration at Current Controlled Trials on the German Clinical Trials Register (http://www.drks.de) ensure both the validity of the methodical procedure as well as the transparency of the whole trial for the scientific community and is a prerequisite for future publications in peer-reviewed journals. A comprehensive independent monitoring strategy including on-site visits and frequent communication according to the standard operating procedure of the Coordination Center for Clinical Trials, Heidelberg will assure data quality and security of patients. In consideration of reducing experimental biases, data collectors of each center (rating staff) are blinded in this study and separated from therapists or other intervention related work. Thus, they will have no knowledge about individual group assignment. Both intervention programs will be implemented by trained therapists with clinical background. Using intention-to-treat-analyses and per-protocol-analyses, the study represents a conservative and clinically useful strategy of data analysis.

Potential study design limitations should also be discussed. A common limitation of controlled trials is the strict inclusion and exclusion criteria. Severe depression is a common comorbidity among persons with ASD. However, these patients cannot take part in the study. The nature of the computer-based SCOTT&EVA intervention makes it necessary to exclude adults with a former or present gambling disorder.

The participation in the FASTER group psychotherapy depends on pragmatic aspects such as the travel distance to the study centers and if offered times of therapy settings match with the working time.

Another limitation is the time-consuming aspect of this trial, which includes a large number of questionnaires, assessments, homework, and weekly therapeutic group sessions or training tasks. Only those patients who can spend this time and effort are able to participate.

The high prevalence rate and the undersupply of treatments in adults with ASD are two important needs for action. This is the first large interventional randomized controlled trial in Germany to target the core symptoms of high-functioning ASD in adults.

## Trial status

Study protocol version 1.94 has been submitted to German Clinical Trials Register (DRKS). Due to the delay caused by the SARS-CoV-2 pandemic, recruitment will start in March 2021. Last patient out has been planned 28 months after first patient inclusion.

### Authors’ contributions {31b}

LTvE and TF conceived the study. LTvE, TF, and UMS initiated the study design. TF, MK, ID, and CK helped with implementation. MK provided statistical expertise in clinical trial design. All authors contributed to refinement of the study protocol and approved the final manuscript.

## Abbreviations

AE: Adverse event
APP: Adult Psychiatry and Psychotherapy
ASD: Autism spectrum disorder
BDSG: Bundesdatenschutzgesetz (Federal Data Protection Law)
CRF: Case report form
CV: Curriculum vitae
DBL: Data Base Lock
DFG: Deutsche Forschungsgemeinschaft (German Research Foundation)
DSM-5: Diagnostic and Statistical Manual of Mental Disorders, 5th Edition
DSMC: Data Safety and Monitoring Committee
EC: Ethics Committee
FASTER: Freiburg Asperger-specific therapy for adults, Freiburger AspergerspezifischeTherapie für ERwachsene
FPI: First patient in
GCP: Good Clinical Practice
GCP-V: Good Clinical Practice Ordinance (GCP-Verordnung)
ICD-10: The International Statistical Classification of Diseases and Related Health Problems, 10th revision
ICH: International Council on Harmonisation of Technical Requirements for Registration of Pharmaceuticals for Human Use
ICH GCP: ICH harmonized tripartite guideline on GCP
IIT: Investigator Initiated Trial
IMBI: Institute of Medical Biometry and Informatics, Heidelberg
ISF: Investigator Site File
ISRCTN: International Standard Randomized Controlled Trial Number
ITT: Intention to treat
KKS: Koordinierungszentrum für Klinische Studien Heidelberg (Coordination Center for Clinical Trials)
LPI: Last patient in
LPO: Last patient out
MMRM: Mixed model repeated measures
n.a.: Not applicable
PCI: Project Coordinating Investigator
PP: Per-protocol
SAE: Serious adverse event
SAP: Statistical analysis plan
SAS: Statistical Analysis Software
SCOTT&EVA: Social COgnition Training Tool & Understanding and Expressing Emotions (German: “Emotionen Verstehen und Ausdrücken”)
SOP: Standard Operating Procedure
TAU: Treatment as usual
TMF: Trial Master File
WHO: World Health Organization

## Abbreviations of questionnaires and other assessments

ADOS-2: Autism Diagnostic Observation Scale, Version 2
ADHS-SB: Attention Deficit Hyperactivity Disorder, self-report
AQ50: Autism Quotient, 50 items
BDI-II: Beck Depression Inventory, Version 2
CFT 20-R: Culture Fair Intelligence Test, Revised
EQ40: Cambridge Behavioural Scale, Empathy Quotient, 40 items
GAF: Global Assessment of Functioning
FEL: Freiburg Events List
FLZ^M^: Life Satisfaction Questionnaire
MET: Multifaceted Empathy Test, computer test
MWT-B: Mehrfach Wahl-Wortschatz-Intelligenz-Test (Muliple choice vocabulary intelligence test)
MSWS: Multidimensional Self-worth Scale
NEO-FFI: Neo Five-Factor-Inventory
SASKO: Questionnaire for social anxiety and social skills deficits
SCID-5-CV: Structured Clinical Interview for DSM-5 for clinical diseases
SCID-5-PD: Structured Clinical Interview for DSM-5 for personality disorders
SRS-A: Social Responsiveness Scale, adult form self-report, parent / spouse / other
SRS-A mod.: Social Responsiveness Scale, adult form self-report, parent / spouse / other, modified version
TAS-26: Toronto Alexithymia Scale-26
WHOQOL-Bref: WHO Quality of Life, short form
WURS-K: Wender-Utah Rating Scale, short form
